# The nature of the self: Neural analyses and heritability estimates of self‐evaluations in middle childhood

**DOI:** 10.1002/hbm.25641

**Published:** 2021-09-03

**Authors:** Lina van Drunen, Simone Dobbelaar, Renske van der Cruijsen, Mara van der Meulen, Michelle Achterberg, Lara M. Wierenga, Eveline A. Crone

**Affiliations:** ^1^ Leiden Consortium of Individual Development (L‐CID) Leiden The Netherlands; ^2^ School of Social and Behavioral Sciences, Developmental Neuroscience in Society Erasmus University Rotterdam Rotterdam The Netherlands; ^3^ Social and Behavioral Sciences, Developmental and Educational Psychology Institute of Psychology, Leiden University Leiden The Netherlands; ^4^ Leiden Institute for Brain and Cognition (LIBC) Leiden The Netherlands

**Keywords:** child, genetic models, magnetic resonance imaging, self‐concept, social environment, twins

## Abstract

How neural correlates of self‐concept are influenced by environmental versus genetic factors is currently not fully understood. We investigated heritability estimates of behavioral and neural correlates of self‐concept in middle childhood since this phase is an important time window for taking on new social roles in academic and social contexts. To do so, a validated self‐concept fMRI task was applied in a twin sample of 345 participants aged between 7 and 9 years. In the self‐concept condition, participants were asked to indicate whether academic and social traits applied to them whereas the control condition required trait categorization. The self‐processing activation analyses (*n* = 234) revealed stronger medial prefrontal cortex (mPFC) activation for self than for control conditions. This effect was more pronounced for social‐self than academic self‐traits, whereas stronger dorsolateral prefrontal cortex (DLPFC) activation was observed for academic versus social self‐evaluations. Behavioral genetic modeling (166 complete twin pairs) revealed that 25–52% of the variation in academic self‐evaluations was explained by genetic factors, whereas 16–49% of the variation in social self‐evaluations was explained by shared environmental factors. Neural genetic modeling (91 complete twin pairs) for variation in mPFC and anterior prefrontal cortex (PFC) activation for academic self‐evaluations confirmed genetic and unique environmental influences, whereas anterior PFC activation for social self‐evaluations was additionally influenced by shared environmental influences. This indicates that environmental context possibly has a larger impact on the behavioral and neural correlates of social self‐concept at a young age. This is the first study demonstrating in a young twin sample that self‐concept depends on both genetic and environmental factors, depending on the specific domain.

## Graphical abstract

We investigated heritability estimates of behavioral and neural correlates of self‐concept in middle childhood since this phase is an important time window for taking on new social roles in academic and social contexts. To do so, a validated self‐concept fMRI task was applied in a twin sample of 345 participants aged between 7 and 9 years. This is the first study demonstrating in a young twin sample that self‐concept depends on both genetic and environmental factors, with stronger environmental influences in the social versus academic domain.

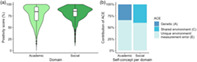

## 1 INTRODUCTION

A unique human ability is the capacity to appreciate oneself as a person with qualities such as social competence and intelligence. The ability to recognize and act on one's feelings and thoughts serves an evolutionary adaptive purpose (Sedikides & Skowronski, 1997). For instance, differentiating feelings and thoughts of oneself from those of others is adaptive for social development (Denny, Kober, Wager, & Ochsner, 2012). Although self‐awareness originates in the first year of life (Rochat & Striano, 2002), self‐concept complexity continually develops from early childhood to adulthood. Increasing cognitive abilities (i.e., perspective taking) and socialization experiences (i.e., taking on new social roles) affect the forming of self‐concept in academic skills and social relationships (Harter, 2012; Marsh & Ayotte, 2003; Muris, Meesters, & van den Berg, 2003). It is still unclear to what extent a differentiated self‐concept is driven by environmental or genetic influences. This study is the first to examine the degree to which variation in behavioral and neural correlates of self‐concept are genetically driven and environmentally influenced at a relatively young age, using a 7–9‐years old twin‐sample (Crone et al., 2020).

Meta‐analyses of functional magnetic resonance imaging (fMRI) studies in adolescents and adults revealed self‐processing activation in cortical midline structures, including ventral and dorsal medial prefrontal cortex (mPFC), anterior and posterior cingulate cortex (ACC/PCC), and medial parietal cortex (Denny et al., 2012; Northoff et al., 2006). Another line of research has identified the mPFC and PCC as regions that are part of the default mode network (DMN; Gusnard, Akbudak, Shulman, & Raichle, 2001). These regions showed greater activation during rest and internal judgments than during goal‐directed tasks (Mason et al., 2007). As such, it is assumed that the DMN possibly supports self‐referential mental activity including unconstraint self‐referential thoughts (Davey, Pujol, & Harrison, 2016; Gusnard & Raichle, 2001). Other studies highlight the importance of the mPFC in mentalizing about the self during adolescence (Amodio & Frith, 2006; Legrand & Ruby, 2009; Pfeifer, Lieberman, & Dapretto, 2007; Qin & Northoff, 2011; van der Meer, Costafreda, Aleman, & David, 2010; van Overwalle, 2011). Additionally, neural regions are differentially involved in processing self‐concept in different domains, such that academic evaluations elicit strong lateral PFC activation whereas social evaluations elicit stronger mPFC activation (Jankowski, Moore, Merchant, Kahn, & Pfeifer, 2014; van der Cruijsen, Peters, & Crone, 2017; van der Cruijsen, Peters, van der Aar, & Crone, 2018). The mPFC thus appears to have an essential role in thinking about ourselves (Denny et al., 2012; Lieberman, Straccia, Meyer, Du, & Tan, 2019), with separable contributions for academic and social self‐evaluations (Jankowski et al., 2014). Only few studies investigated neural processing of self‐concept in children, showing increased mPFC activation in 9–10‐year‐old children during self‐evaluations when compared to adults (Pfeifer et al., 2007) and increased cortical midline activation during direct self‐evaluations in 10–13‐year‐olds (Barendse et al., 2020) and 11–14‐year‐olds (Jankowski et al., 2014). Furthermore, ACC activation was observed during self versus close‐other processing with increasing age (7–13‐years; Ray et al., 2009). However, no prior study examined neural activity in young children while they evaluate oneself in different domains, similar to what has been examined in adolescents and adults (van der Cruijsen et al., 2017, 2018).

Genetic and environmental influences on self‐concept can best be studied in middle childhood, as this period marks a shift in cognition and social behavior (DelGiudice, 2018) in relation to self‐concept. In early childhood, children compare themselves to themselves in the past (temporal comparisons), whereas in middle childhood, children start to engage in social comparison (Harter, 2012) which may lead to increased social environmental influences affecting their self‐concept. Still, self‐concept in middle childhood is relatively understudied. A study in 11–12‐year‐old female preadolescents reported that 30% of variance in self‐concept regardless of domain was explained by genetic factors. The remaining variance accounted primarily for unique environmental factors/measurement error (Hur, McGue, & Iacono, 1998). A meta‐analysis on self‐esteem in adolescents and adults observed minimal effects of shared environment (10%), and substantial effects of genetic influences (30–50%; Neiss, Sedikides, & Stevenson, 2002). Taken together, these studies led to the question whether behavioral and neural markers of self‐evaluations are accounted for by genetic or environmental factors in childhood.

Here, we used genetic modeling in young twins to estimate genetic and environmental (shared/unique) influences on self‐concept. We aimed to investigate: (a) How are domain‐specific self‐evaluations related to neural correlates in 7–9‐year‐old children using fMRI? (b) What are the influences of genetic factors, shared environment, and unique environment/measurement error on the behavioral and neural correlates of self‐concept? We differentiated between positively and negatively valenced traits as prior studies demonstrated that positive traits result in stronger mPFC activity (van der Cruijsen et al., 2018). We hypothesized that self‐evaluations are associated with mPFC activation with separable contributions to academic and social domains (Denny et al., 2012; Northoff et al., 2006; van der Cruijsen et al., 2018; van der Meer et al., 2010). We expected that genetic and environmental factors would both contribute to academic and social self‐concept on a behavioral level (Harter, 2012; Hur et al., 1998). This is the first study to examine the heritability of neural responses to self‐concept. A prior heritability study on structural brain measures in relation to prosocial behavior reported that a mixed nature of genetic and environmental factors influenced the mPFC region in 7–9‐year‐old children (van der Meulen, Wierenga, Achterberg, Drenth, & van IJzendoorn, M. H.,, & Crone, E. A., 2020). Additional fMRI studies revealed small genetic influences on neural activity of social rejection (Achterberg, Van Duijvenvoorde, van der Meulen, Bakermans‐Kranenburg, & Crone, 2018) and prosocial behavior (van der Cruijsen et al., 2018) in children in the same age‐range. Therefore, we expect that the current design will allow us to unravel genetic and environmental (shared/unique) influences on neural self‐concept.

## 2 METHODS

### 2.1 Participants

Participants took part in the early childhood cohort of the Leiden Consortium on Individual Development (L‐CID) study and participated in wave 5 (Crone et al., 2020), in which data of the self‐concept fMRI paradigm was exclusively collected. The participants were twins born between 2010 and 2011 and recruited through municipal registries (Euser et al., 2016). DNA analyses were performed to determine zygosity through buccal cell samples collected via mouth swabs (Whatman Sterile Omni Swab).

In total, 345 (of 360) participants were included in the behavioral analyses of the means (mean age 7.53, ranging from 6 to 9; 46% boys). This sample included 166 complete twin‐pairs (61% MZ; see Table 1). Of these 345 participants, 234 participants had MRI data that passed inclusion criteria (mean age 7.56, ranging from 7 to 9; 45% boys). In total, 15 (of 360) participants were excluded from all analyses due to a technical error in collecting behavioral data, 62 participants were excluded from MRI analyses because of incomplete MRI data, anxiety, nonremovable braces, and no parental consent for MRI participation, and 49 participants were excluded from MRI analyses because of movement beyond 3 mm. This MRI sample included 91 complete twin‐pairs (59% MZ; see Table 1). Prior to the current study of the L‐CID project (Crone et al., 2020), 61 (35%) randomly selected families received an intervention aimed to enhance parental sensitivity and sensitive discipline strategies of the primary caregiver, named VIPP‐SD (Euser et al., 2016) as part of a randomized control intervention design. We controlled for group condition (control or intervention group) in our behavioral analyses, because the emphasis of the current study was not on the intervention. In addition, intelligence quotient (IQ) was estimated at wave 4 with “Picture completion” as subset of the Wechsler Preschool and Primary Scale of Intelligence—Third Edition (WPPSI‐III, Preschool, 2002). Estimated Performance IQ's (PIQ) of the participants were ranged normally between 65 and 135. We controlled for PIQ in our behavioral analyses. No significant correlations were observed between PIQ and self‐concept.

**TABLE 1:** Demographic characteristics

	Behavioral sample	Behavioral heritability sample	MRI sample	MRI heritability sample
*N*	345	332	234	182
Boys	46%	46%	45%	43%
Left‐handed	12%	12%	11%	10%
Age (*SD*)	7.53 (0.59)	7.54 (0.60)	7.56 (0.58)	7.56 (0.56)
Range	6–9	6–9	7–9	7–9
Complete twin pairs	166	166	91	91
Monozygotic	61%	61%	59%	60%
Median IQ^a^	105	105	105	105
IQ range	65–135	65–135	65–135	65–135

Abbreviations: *N*, number of participants; SD, standard deviation.

^a^ At wave 4.

The study and procedures were approved by the Dutch Central Committee on Research Involving Human Subjects (CCMO). Both parents signed the informed consent before they were included in the study. All pairs of twins had a shared environment at home, reported normal or corrected‐to‐normal vision, and reported no neurological or psychiatric impairments.

### 2.2 Experimental design

**FIGURE 1:**
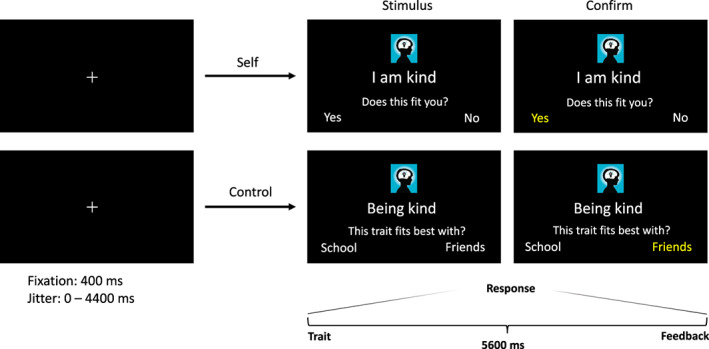
Example of a trial in the self‐ and control condition. Each trial started with a jittered duration between 0 and 4400 ms, followed by a 400 ms fixation cross on a black screen. Next, the stimulus of either the self‐condition or control‐condition was presented. In the self‐ condition, the individuals were asked to indicate whether the academic and social traits applied to them by answering “*Yes*” or “*No*.” In the control‐condition, the individuals were asked to categorize the trait sentence into “*School*” or “*Friends*.” A screen with the phrase *“Too late!*” was shown for another 1000 ms when the individual failed to answer within the given time period. The self‐and control stimuli were always shown for 5600 ms before the next trial started

The participants completed an fMRI task in which they read and listened to short sentences that were presented on a screen in the MRI scanner, describing either positively or negatively valenced traits in the social and academic domain (see Figure 1). Van der Cruijsen et al. (2018) validated the fMRI task by correlating the academic and social domains to subscales of the Dutch version of the Self Perception Profile for Adolescents (SPPA; Harter, 1988). Since the fMRI task in the study of Van der Cruijsen et al. (2018) was based on participants aged between 11 and 21 years old, small adjustments were made in the task of this study to make it feasible for children aged between 7 and 9 years old. Adjustments that were made are (a) “yes” and “no” answers instead of 1–4 scales to ask the children to what extent the self‐evaluations applied to them, (b) inclusion of two self‐concept domains (academic, social) instead of three (academic, social, physical), and (c) text‐edits of self‐trait sentences to sentences that were more age appropriate when necessary. These adjustments, together with the stimulus presentation and response recording, were made and controlled with the use of the E‐Prime software (Schneider, Eschman, & Zuccolotto, 2002; version 3.0).

The task consisted of a self‐condition and a control‐condition. In the self‐condition, participants were asked to indicate whether the academic and social traits applied to them by answering “*Yes*” or “*No*.” Participants responded to 40 short written and read sentences, such as “*I am kind*” (positive social trait) or “*I am unintelligent*” (negative academic trait), by pressing the left button for “*Yes*” and right button for “*No*” or vice versa, counter balanced across families. Twenty sentences were displayed for each domain (academic and social traits) of which 10 were positively valenced and 10 were negatively valenced. The control‐condition consisted of a total of 20 short sentences of which 10 with a positive value and 10 with a negative value. They were asked to categorize trait sentences according to two categories: “*School*” and “*Friends*.” An example of a trait sentence in the control condition is *“Being smart, this trait fits best with?”* (academic trait). The self‐condition and control‐condition were completed in separate runs. The order of the self‐and control‐condition was counterbalanced across twin‐pairs. A list with all the trait sentences is presented in DataverseNL.

All trials in both conditions were completed in a pseudorandomized order. Each trial began with a 400 ms fixation cross, followed by a stimulus screen including the trait sentence and the response options (“*Yes*” or “*No*”) which were presented for 5600 ms. Participants could respond to the trait within this period of time. The answer they chose turned yellow for the remaining stimulus time to guarantee participants their choice was registered in the task. However, if the participant did not respond within the 5600 ms, the phrase “*Too late!”* was presented for another 1000 ms. These trials (4%) were modeled as a separate regressor of no interest. The optimal jitter timing and the order of the trials were computed with Optseq 2 (Dale, 1999), ranging between 0 and 4400 ms.

### 2.3 fMRI data acquisition

The MRI scans were acquired on a Philips Ingenia 3.0 Tesla MRI system with a standard whole‐head coil. To restrict head motion, foam inserts were applied to the children's head. The full scan protocol took approximately 40 min including two Pacs Survey scans, high‐resolution T1‐weighted and T2‐weighted scans, two fMRI tasks and finally a resting‐state fMRI scan. Each participant underwent the scans in the same order. The fMRI task‐stimuli were displayed on a screen, attached in the magnet bore, which could be viewed through a small mirror that was placed on the head coil. First, a high‐resolution 3D T1‐weigthed scan was obtained for anatomical reference (repetition time [RT] = 9.72 ms; echo time (TE) = 4.95 ms; 140 slices; field of view (FOV) = 224 (ap) × 177 (rl) × 168 (fh); flip angle (FA) = 8°; voxel size 0.875 × 0.875 × 0.875 mm). Subsequently, the functional scans were acquired in two runs using a T2*‐weighted echo‐planar imaging (EPI) sequence, in which the first two volumes were discarded to account for T1 saturation effects (TR = 2.2 s; TE = 30 ms; sequential acquisition; 37 slices; field of view (FOV) = 220 (ap) × 220 (rl)× 111.65 (fh); FA = 80°; voxel size = 2.75× 2.75× 2.75 mm). Participants were excluded when excessive head motion was observed, defined as >3 mm motion in any direction (*x*, *y*, *z*) in one or two of the blocks of the fMRI task (Achterberg & van der Meulen, 2019).

### 2.4 fMRI preprocessing

SPM8 (Welcome Trust Centre for Neuroimaging, London) was used to preprocess and analyze the MRI data. The functional images were adjusted for slice‐timing acquisition and rigid body movement. Furthermore, functional volumes were spatially normalized to the T1 templates. A 12‐parameter affine transformation was used for the normalization in addition with a nonlinear transformation containing cosine basis functions, followed by resampling the volumes to 3 × 3 × 3 mm^3^ voxels. Templates were based on the MNI305 stereotaxic space (Cocosco, Kollokian, Kwan, Pike, & Evans, 1997). Finally, all volumes were spatially smoothed with an isotropic Gaussian Kernel with 6 mm full‐width at half‐maximum (FMHM).

### 2.5 First level analyses

The task effects on the individual participant's data were estimated using a general linear model with SPM8. The time series during the fMRI task were modeled as zero duration events convolved with the hemodynamic response function (HRF) and are defined by their specific onset and duration. The invalid trials, in which the participants failed to respond within the given time, were modeled separately as events of no interest. The modeled events of interest were framed as “Academic‐Positive,” “Academic‐Negative,” “Social‐Positive,” “Social‐Negative,” and “Control”. These aforementioned trials of interest were used as regressors in the general linear model together with a set of cosine functions that high‐pass filtered the data. The least‐squares parameter estimates of height of the best‐fitting canonical HRF for each condition were obtained in the following pair‐wise contrasts: *Self* > *Control*, *Negative Self* > *Positive Self* (and vice versa), *Academic* > *Social* (and vice versa). Finally, the resulting contrast images, calculated on an individual level, were submitted to higher‐level group analyses.

### 2.6 Second level group analyses

In order to investigate our aims, we performed two group analyses. First, all self‐condition events were compared to the control‐condition events by using a one sample *t*‐test to analyze the contrast *Self > Control*. In the second analysis, a full factorial whole‐brain 2 (valence: negative, positive) × 2 (domain: academic, social) ANOVA was performed. We checked whether we could observe main effects of valence and/or domain to subsequently explore the valence‐ and domain‐specific neural activity within the contrasts *Negative Self > Positive Self* (and vice versa) and *Academic > Social* (and vice versa). For all analyses, the false discovery rate (FDR) cluster level correction (*p* < .05) was applied at an initial uncorrected threshold of *p* < .001, as implemented in SPM8. Of note, we compared the whole brain analyses of the complete MRI sample (*n* = 234) and an MRI subsample (*n* = 143; incomplete twin pairs: children whose twin was excluded from fMRI analyses) to check for possible twin‐pair effects. We observed similar whole brain activation in both samples, arguing against the possibility that the ROI selection is biased towards one sample.

### 2.7 Region of interest analyses

Regions of interest (ROIs) were selected for subsequent heritability analyses. We used two approaches to determine heritability estimates of neural processing of self‐evaluations. Based on prior child, adolescent, and adult studies, the mPFC appears to have a key role in evaluating oneself (Jankowski et al., 2014; Legrand & Ruby, 2009; Pfeifer et al., 2007; van der Cruijsen et al., 2017, 2018; van der Meer et al., 2010; van Overwalle, 2011). Therefore, first an independent ROI approach of the mPFC region of Denny et al. (2012) (*x* = −6, *y* = 50, *z* = 4) was used since they performed a meta‐analysis of 107 neuroimaging studies on self‐concept. As such, they provide reliable coordinates of the mPFC region based on a large number of scans. Furthermore, the mPFC region (Denny et al., 2012) was also used as ROI in the study of van der Cruijsen et al. (2018), on which our fMRI self‐concept paradigm is based. Second, nine ROIs based on the complete MRI sample (*n* = 234) were selected by a data‐driven approach. Clusters of activation from the whole brain group contrasts (Self > Control; Negative Self > Positive Self; Positive Self > Negative Self; Academic > Social; Social > Academic) were extracted to select our ROIs using the MarsBar toolbox (Brett, Anton, Valabregue, & Poline, 2002). The exploratively selected data‐driven ROIs were most commonly described in the previously brain‐behavior literature in relation to self‐evaluations. For the *Self > Control* contrast, we selected the mPFC region (Jankowski et al., 2014; Legrand & Ruby, 2009; Pfeifer et al., 2007; van der Cruijsen et al., 2017, 2018; van der Meer et al., 2010; van Overwalle, 2011). For the contrast *Negative Self > Positive Self*, we selected lateral PFC and mPFC regions, and for the reversed contrast the superior mPFC and the ventral subgenual mPFC regions (Denny et al., 2012; Jankowski et al., 2014; van der Cruijsen et al., 2017, 2018; van der Meer et al., 2010). For the contrast *Academic > Social*, we selected the left and right DLPFC, and for the reversed contrast the mPFC (Moran, Macrae, Heatherton, Wyland, & Kelley, 2006; van der Cruijsen et al., 2017).

### 2.8 Statistical analyses: Behavior

To investigate how the individuals evaluated themselves on positive and negative traits in different domains, we used a linear mixed‐model approach with the nlme package (Yuan et al., 2020; version 3.1‐148) in R (R Core Team, 2015). The effects of valence (negative and positive) and domain (academic and social) on the percentage of times participants choose “*yes*” on trait sentences were assessed. First, data were fitted on the percentages of (a) “*yes*” answers on positive traits of the academic domain, (b) “*yes*” answers on negative traits of the academic domain, (c) “*yes*” answers on positive traits of the social domain and (d) “*yes*” answers on negative traits of the social domain. The variables were computed in R as:

$$\;\sum_{i=1}^{N}i\mathrm{YES}\;\text{domain valence}\;\left(\%\right)=\frac{\sum_{i=1}^{N}i\mathrm{YES}\;\text{domain valence}}{\left(\sum_{i=1}^{N}i\mathrm{YES}\;\text{domain valence}\right)+\sum_{i=1}^{N}i\mathrm{NO}\;\text{domain valence})\;}\times 100$$

Random intercepts per family were used to account for the nesting between twin‐pairs within families (FamilyID). The fixed factors consisted of valence (negative and positive), domain (academic and social) and zygosity, while controlling for IQ and the intervention group (intervention and control). All main effects and the two‐way interactions were obtained (valence × domain, domain × VIPP, and domain × IQ). To investigate how children evaluated themselves, we specified the fitted mixed linear model in R as:

$$\sum_{i=1}^{N}i\mathrm{YES}\;\text{domain valence}\;\left(\%\right)\sim \text{Valence}\times \text{Domain}+\text{Domain}\times \text{VIPP}+\text{Domain}\times \mathrm{IQ}\;+\text{Zygosity}+\left(1|\text{FamilyID}\right)+\varepsilon$$

Last, both negative and positive self‐evaluations in the academic and social domain were combined into two applicability positivity scores, in which a higher score indicated a more positive self‐concept within the domain. The positivity scores, describing self‐concept per domain in the present study, were used as variables for behavioral genetic modeling. These scores were calculated in R as:

$$\sum_{i=1}^{N}\text{iPositivity academic}\;(\%)=\frac{(100-\sum_{i=1}^{N}i\mathrm{YES}\;\text{academic negative}\;\left(\%)\right)+\sum_{i=1}^{N}i\mathrm{YES}\;\text{academic positive}\;\left(\%\right)}{2}$$

$$\sum_{i=1}^{N}\text{iPositivity social}\;\left(\%\right)=\frac{(100-\sum_{i=1}^{N}i\left(\mathrm{YES}\;\text{social negative}\;\left(\%\right)\right)+\sum_{i=1}^{N}i\mathrm{YES}\;\text{social positive}\;\left(\%\right)}{2}$$

### 2.9 Genetic modeling

To investigate the genetic and environmental influences on differences in self‐concept behavior and neural correlates of self‐concept, we first performed within‐twin pair Pearson correlations for each outcome variable, separately for monozygotic (MZ) and dizygotic (DZ) twins (Achterberg et al., 2018; van der Meulen, Steinbeis, Achterberg, van IJzendoorn, M. H, & Crone, 2018). Whenever MZ twins scored a higher correlation than DZ twins, it would indicate an influence of genetic factors. When DZ twins scored a higher correlation than half the MZ correlation or both DZ twins and MZ twins scored high correlation coefficients, this would indicate that shared environment was also an important contributor to the model. We used Fisher r‐to‐z transformations to test whether within‐twin correlations were significantly different for MZ compared to DZ twins. A correlation smaller than 1 of genetic and/or shared environment would indicate a remaining effect of unique environment/measurement error (van der Meulen et al., 2018).

For further testing, a structural equation ACE model with the OpenMX package (Neale et al., 2016; version 2.7.4) was used in R (R Core Team, 2015) to get a more specific estimate of the relative contributions of additive genetic factors (A), shared environmental factors (C) and unique environment/measurement errors (E). The correlation between the shared environment (factor C) was set to 1 for both MZ and DZ twins (share 100% of their shared environment at home), while the correlation between the genetic factor (A) was set to 1 for MZ twins (share 100% of their genes) and to 0.5 for DZ twins (share approximately 50% of their genes similar to siblings). The last factor, unique environmental influences and measurement error, was freely estimated (Neale et al., 2016).

### 2.10 Exploratory brain‐behavior associations

To test for possible brain‐behavior associations, the behavioral positivity scores in the academic and social domain were correlated to the activity of three related ROIs using several Pearson correlations. The ROIs were extracted from the *Negative Self > Positive Self* contrast (left lateral PFC, right anterior PFC, and dorsal mPFC). We included this contrast to test the hypothesis that negative self‐concept showed increased activity in the PFC and dorsal mPFC compared to positive self‐concept, indicating additional reactions of active emotion regulation (Silvers et al., 2016; Silvers & Moreira, 2019).

## 3 RESULTS

We detected outliers by transforming the raw data into standardized *z* values. Z scores outside the 99.9% range of the Z‐distribution were defined as outliers (−3.29 < x > 3.29). These scores were excluded from further behavioral and neural analyses.

### 3.1 Behavioral results: Means

**FIGURE 2:**
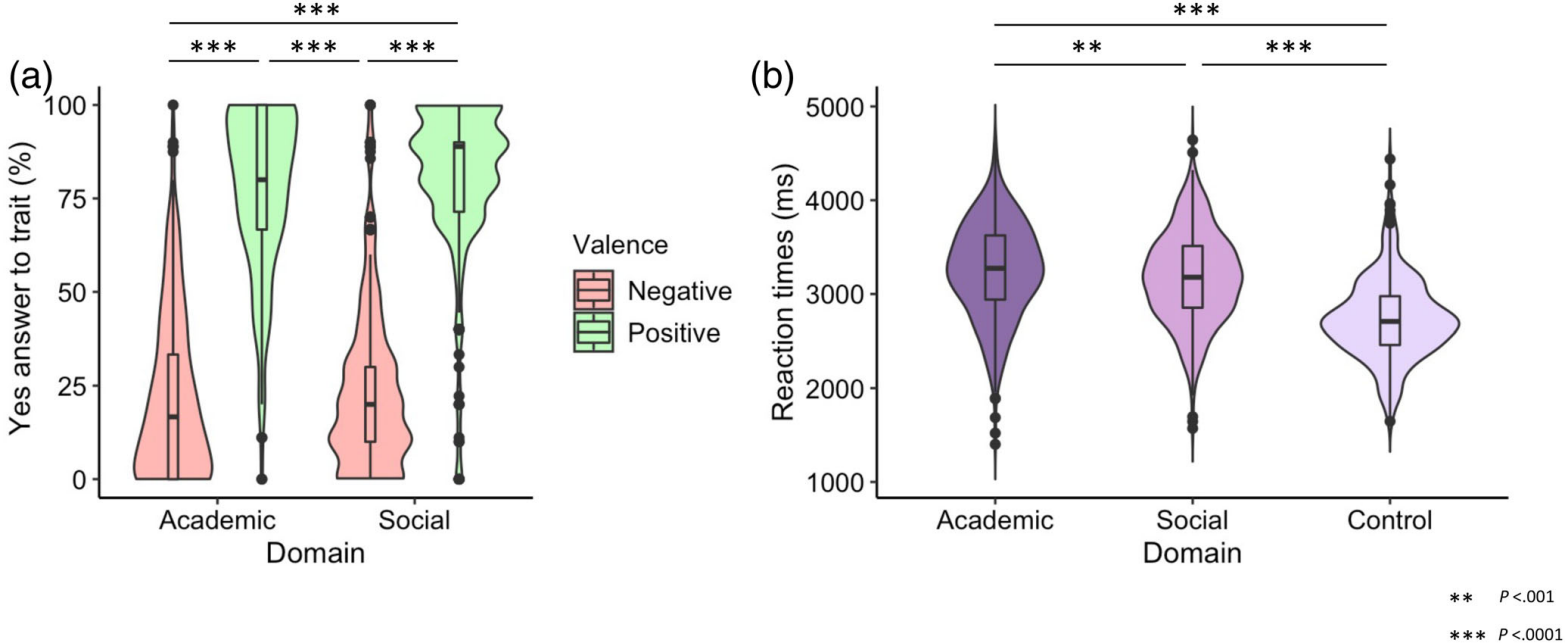
(a) “Yes” ratings for self‐evaluations in the academic and social domain, separated for positive and negative valence trials. Children rated themselves more often positively than negatively in both domains. (b) Reaction times (RTs) of self‐evaluations in the academic, social, and control condition. Children reacted slower to academic self‐evaluations than to social self‐evaluations and control condition

**FIGURE 3:**
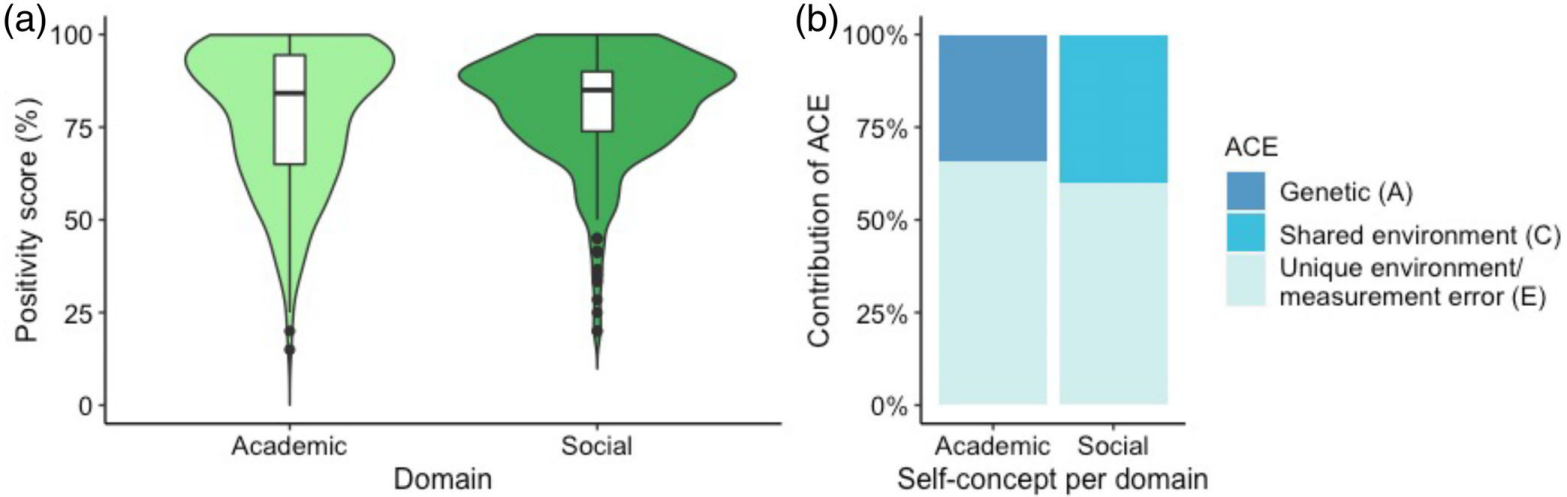
The positivity scores as indicators of self‐concept per domain and heritability estimates. (a) A higher positivity score indicated a more positive self‐concept within the domain. No significant difference in positivity ratings was observed between the academic and social domain. (b) Behavioral genetic modeling revealed that academic self‐concept depends on genetic factors (A), whereas social self‐concept depends on shared environmental influences (C)

Percentage of self‐evaluations per domain and valence of 345 participants are presented in Figure 2. The linear mixed model for the percentage of self‐evaluations showed a significant main effect of valence (*β* = 55.10, *SE* = 1.77, *F*(1,1047) = 31.18, *p* < .001). This model revealed that the percentage “*yes”* to positive trait sentences (*M* = 79%, *SE* = 0.91, 95% CI [77%, 81%]) was significantly higher than the percentage “*yes”* to negative trait sentences (*M* = 23%, *SE* = 0.91, 95% CI [21%, 25%]). In summary, these results showed a positivity bias in children with higher positivity ratings compared to negativity ratings across both domains, see Figure 2a. The positivity scores, indicating academic and social self‐concept in the present study, were calculated and are shown in Figure 3a. No difference was observed between the academic and social positivity scores. These positivity scores were used for genetic modeling on a behavioral level.

The linear mixed model for the reaction time (RT) of self‐evaluations showed a significant main effect of domain (*F*(2,1544) = 288.12, *p* < .0001). Pairwise comparisons revealed that the RT of academic self‐trait sentences (*M* = 3250 ms, *SE* = 29.7, 95% CI [3179, 3322]) was significantly longer than the RT of social self‐trait sentences (*M* = 3176, *SE* = 29.7, 95% CI [3104, 3248], *p* = .0001) and then the RT of the control condition (*M* = 2731 ms, *SE* = 32.4, 95% CI [2653, 2810], *p* < .0001). Note that there was also a significant difference in RT between social domain and control condition. In summary, the participants reacted slower to academic self‐traits compared to social self‐traits and the control condition, see Figure 2b.

### 3.2 Behavioral results: Heritability estimates

To explore the genetic and shared environmental influences on the academic and social positivity scores, we first performed within‐twin pair Pearson correlations for each outcome variable, separately for MZ (*n* = 102 and DZ (*n* = 64) complete twin pairs (see Table 1 for behavioral heritability sample). For academic self‐concept, we found significant positive associations within MZ twins, whereas for social self‐concept, we found positive associations within MZ and DZ twins. The within‐twin correlation was significantly different for MZ compared to DZ twins for academic self‐concept (see Table 2).

**TABLE 2:** Contributions of ACE in behavioral genetic modeling for academic and social self‐concept

Outcome variables		MZ	DZ	Z	Model	A^2^	C^2^	E^2^
Positivity academic	*r*	.36	.10	2.43^**^	*ACE*	0.34	0.00	0.66
	*p*	<.001	.46		*95% CI*	[0.16, 0.49]	[*NA*, 0.32]	[0.51, 0.84]
Positivity social	*r*	.41	.33	.82	*ACE*	0.00	0.40	0.60
	*p*	<.001	<.05		*95% CI*	[0.00, 0.51]	[0.25, 0.52]	[0.47, 0.75]

Abbreviations: A, additive genetic; C, shared environment; CI, confidence interval; DZ, dizygotic; E, unique environment/measurement error; MZ, monozygotic; *NA*, not available, model not able to calculate the CI; *p*, *p*‐value of significance, *r*, Pearson correlation, Z, Test statistic *z*, significant Z‐scores indicate significant difference between MZ and DZ correlations.

^**^ *p* < .01.

In the second step, we analyzed the twin effects formally in the structural equation ACE model for each condition to get an estimate of the shared genetic factors (A), shared environment factors (C) and unique environmental factors and measurement errors (E). Behavioral genetic analyses revealed that 34% of the variation in academic positivity (95% confidence interval [CI]: [16%, 49%]) was explained by genetic factors. All other variation was explained by unique environment/measurement error (E = 66%, CI: [51%, 84%]). Furthermore, 40% of the variation of social positivity was explained by shared environmental influences (CI: [25%, 52%]). The remaining variation was accounted primarily for by unique environment/measurement error (E = 60%, CI: [47%, 75%]), see Figure 3b and Table 2.

### 3.3 fMRI results

#### 3.3.1 fMRI results: General self‐evaluations

**FIGURE 4:**
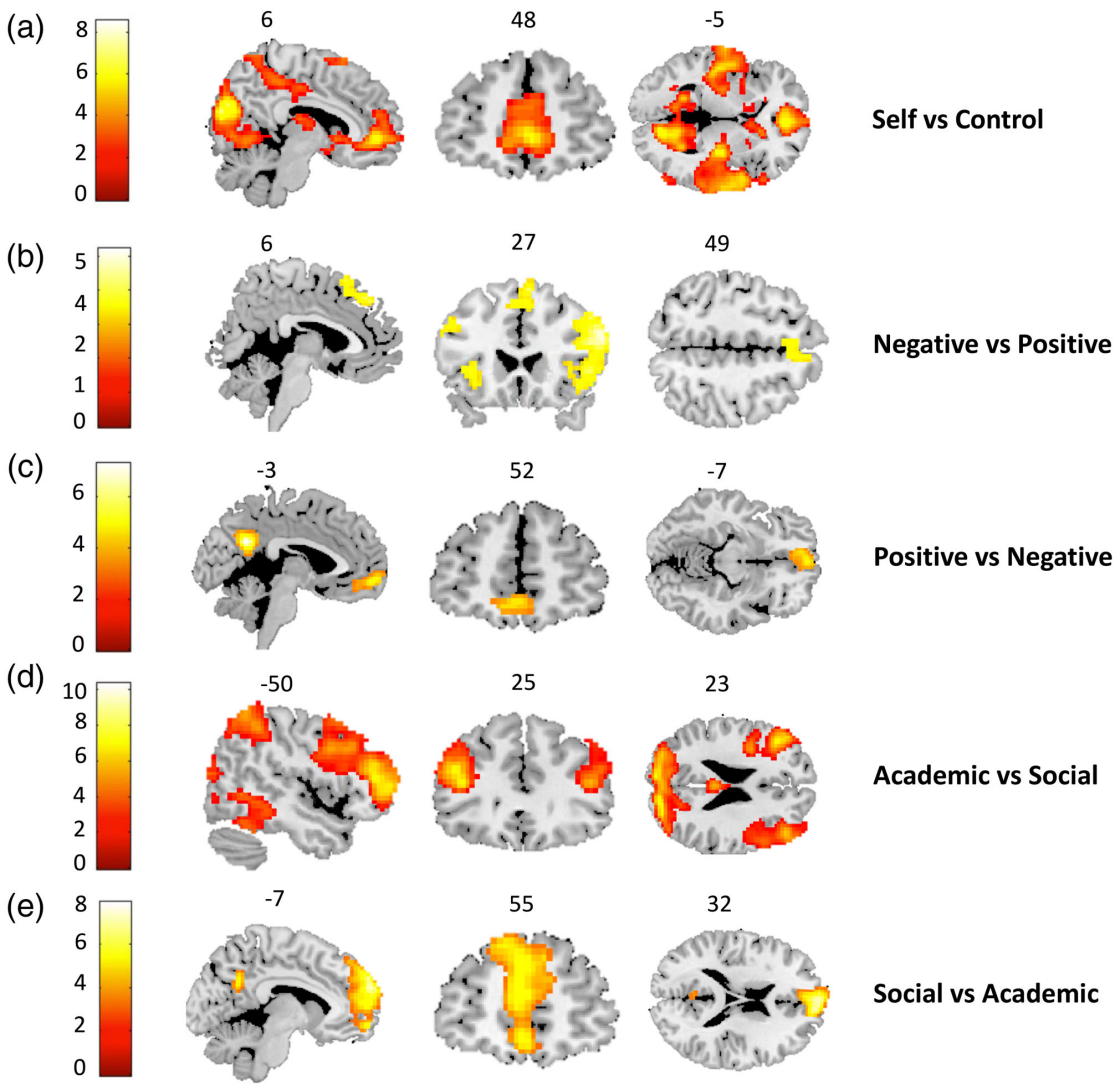
Whole brain results. (a) Activity for self‐evaluations in *Self* versus *Control* contrast with specific activation in mPFC. (b) Activity for self‐evaluations in *Negative Self* versus *Positive Self* contrast with specific activation in dorsal mPFC, right anterior PFC, and left lateral PFC. (c) Activity for self‐evaluations in *Positive Self* versus *Negative Self* contrast with specific activation in PCC and ventral subgenual mPFC. (d) Activity for self‐evaluations in *Academic* versus *Social* contrast with specific activation in bilateral DLPFC. (e) Activity for self‐evaluations in *Social* versus *Academic* contrast with specific activation in mPFC. For all analyses, FDR cluster level correction (*p* < .05) was applied and a cluster‐defining threshold of *p* < .001

To test the first aim of this study, we explored the neural correlates of the participants' self‐concept. First, we examined the neural activity for the *Self > Control* contrast by conducting a whole‐brain one‐sample *t*‐test to examine whether participants activated specific brain regions stronger during self‐evaluations compared to control trials. This contrast resulted in several significant clusters, including (rostral) mPFC (see Figure 4a for an overview of the whole brain results and Table 3 for the related clusters and peaks). The reversed contrast did not result in significant activations.

**TABLE 3:** Regions activated during Self versus Control, Negative Self versus Positive Self (and vice versa) and Academic versus Social (and vice versa)

Region	Cluster size	*p*_FDR_ cluster	*T*	*x*	*y*	*z*
**Self > Control**						
Left cuneus	7657	<.001	8.53	0	−85	28
Left cuneus			7.87	−9	−82	28
Right cuneus			7.73	12	−79	31
Right SMA	139	<.05	5.11	12	11	67
Right SMA			4.53	6	20	67
Right superior frontal gyrus			4.35	21	11	52
Right middle cingulate cortex	583	<.001	4.89	3	−34	49
Right precuneus			4.67	3	−43	55
Left middle cingulate cortex			4.49	−9	−16	40
**Negative > Positive**						
Left middle frontal gyrus	679	<.001	5.22	−51	29	34
Left inferior frontal gyrus			5.05	−42	35	16
Left middle frontal gyrus			4.93	−45	35	28
Right middle frontal gyrus	1191	<.001	5.22	30	62	22
Right inferior frontal gyrus			5.04	51	23	25
Right superior medial gyrus	178	<.01	4.66	6	35	46
Right SMA			4.60	6	20	61
Left SMA			4.10	−6	17	49
**Positive > Negative**						
Left precuneus	301	<.001	7.32	−6	−55	28
Left angular gyrus	133	<.05	5.88	−51	−67	37
Left middle occipital gyrus			3.37	−42	−76	34
Left middle orbital gyrus	199	<.001	5.40	0	59	−8
**Academic > Social**						
Left inferior temporal gyrus	17363	<.001	10.35	−57	−55	08
Right lingual gyrus			9.65	15	−76	−8
Left lingual gyrus			9.11	−12	−82	−14
Right middle orbital gyrus	174	<.001	6.62	27	38	−14
Right olfactory cortex			4.50	15	14	−17
Left superior frontal gyrus	154	<0.01	5.25	−30	−1	67
Left middle frontal gyrus			4.70	−30	8	61
**Social > Academic**						
Left middle orbital gyrus	1309	<.001	7.95	−3	62	−11
Left superior medial gyrus			7.78	−6	62	25
Left anterior cingulate cortex			6.95	−3	62	13
Left precuneus	186	<.01	7.29	0	−58	31
Left middle temporal gyrus	186	<.01	6.52	−60	−13	−17
Left middle temporal gyrus			5.92	−48	2	−32
Left angular gyrus	154	<.01	6.52	−45	−61	28

*Note*: The MNI coordinates (*x*, *y*, *z*) are reported at a cluster‐corrected threshold of *p* < .05 FDR‐corrected, with a primary threshold of *p* < .001 implemented in SPM8.

Abbreviations: SMA, supplementary motor area; T, *T*‐value of *T*‐test.

#### 3.3.2 fMRI results: Valence‐ and domain‐specific self‐evaluations

Next, we examined the neural activity for the *Negative Self* versus *Positive Self* and *Academic* versus *Social* contrasts by conducting a whole‐brain full factorial ANOVA to test for valence‐ and domain‐specific self‐evaluations in brain regions. This analysis showed a main effect of valence and domain.

For further analyses, we explored the main effect for valence in more detail and compared activity for the contrast *Negative Self > Positive Self* (and the reversed contrast). The *Negative Self > Positive Self* contrast resulted in activation in left lateral PFC, right anterior PFC and dorsal mPFC. The reversed contrast *Positive Self* > *Negative Self* showed activation in PCC and ventral subgenual mPFC for positive relative to negative evaluations (see Figure 4b,c for an overview of the whole brain results and Table 3 for the related clusters and peaks).

In addition, we explored the main effect for domain in more detail and compared activity for the contrast *Academic > Social* (and the reversed contrast). The *Academic > Social* contrast analysis resulted in stronger activation in bilateral DLPFC. The reversed contrast *Social* > *Academic* resulted in significant activation in mPFC (see Figure 4d–e for an overview of the whole brain results and Table 3 for the related clusters and peaks).

### 3.4 fMRI results: ROI heritability analyses

**FIGURE 5:**
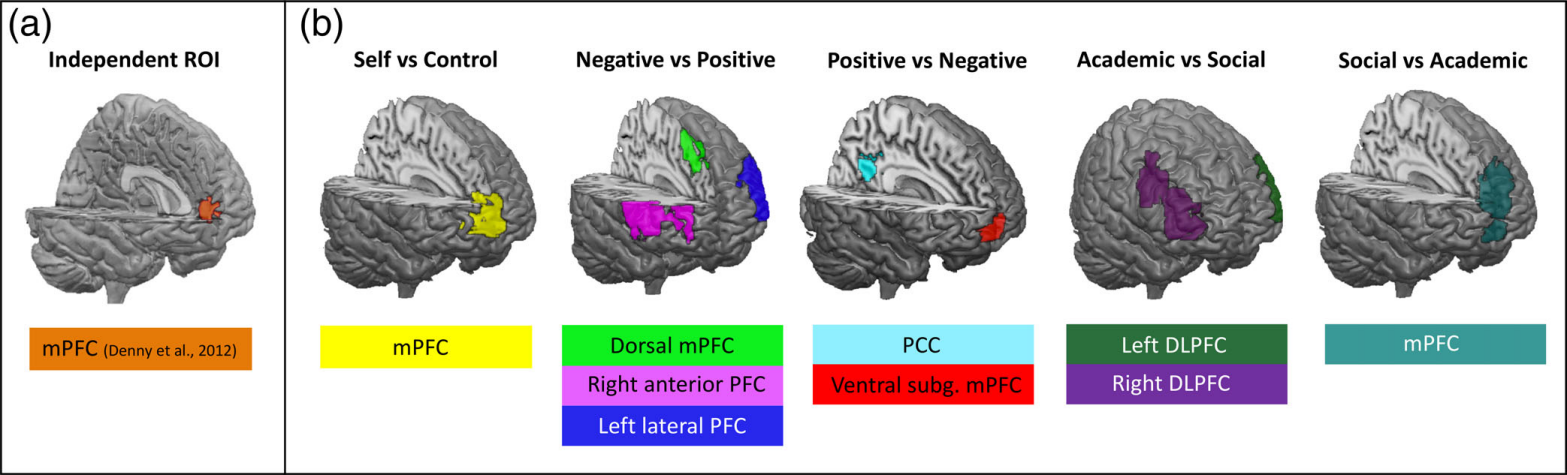
An overview of the ROIs used for neural genetic modeling. (a) The independent mPFC ROI (Denny et al., 2012) and (b) the nine exploratively selected data‐driven ROIs for the *Self* versus *Control*, *Negative Self* versus *Positive Self*, *Positive Self* versus *Negative Self, Academic* versus *Social*, and *Social* versus *Academic* contrasts

To investigate the contributions of genetic and environmental influences on differences in brain activity for the contrasts *Self* versus *Control*, *Negative Self* versus *Positive Self* (and vice versa) and *Academic* versus *Social* (and vice versa) , we performed within‐twin pair Pearson correlations for the independent mPFC ROI (Denny et al., 2012) and the nine data‐driven ROIs based on the complete MRI sample (*n* = 234), separately for MZ (*n* = 55) and DZ (*n* = 36) twin pairs (see Figure 5a for an overview of the independent mPFC ROI (Denny et al., 2012) and *5B* for the nine exploratively selected data‐driven ROIs; See Table 4 for the 95% confidence intervals).

**TABLE 4:** Contributions of ACE in neural genetic modeling for academic and social self‐concept

ROI		MZ	DZ	Z	Model	A^2^	C^2^	E^2^
**Self > Control**								
mPFC (Denny et al., 2012) academic	*r*	.19	.04	.99	*ACE*	0.14	0.00	0.86
	*p*	.16	.81		*95% CI*	[0.00, 0.37]	[0.00, 0.30]	[0.63, 1.00]
mPFC (Denny et al., 2012) social	*r*	.10	.30	−1.35	*ACE*	0.00	0.17	0.83
	*p*	.48	.08		*95% CI*	[0.00, 0.43]	[0.00, 0.37]	[0.57, 1.00]
mPFC academic	*r*	<−.01	.15	−.92	*ACE*	0.00	0.07	0.93
	*p*	1.00	.39		*95% CI*	[*NA*, 0.28]	[0.00, 0.27]	[0.72, 1.00]
mPFC social	*r*	−.18	.10	−1.83^*^	*ACE*	0.00	0.00	1.00
	*p*	.18	.62		*95% CI*	[0.00, 0.16]	[*NA*, 0.15]	[0.85, *NA*]
**Negative self > Positive self**								
Left lateral PFC academic	*r*	.02	.11	−.59	*ACE*	0.00	0.08	0.92
	*p*	.86	.53		*95% CI*	[0.00, 0.37]	[0.00, 0.28]	[0.63, 1.00]
Left lateral PFC social	*r*	−.12	.31	−2.85^*^	*ACE*	0.00	0.09	0.91
	*p*	.40	.06		*95% CI*	[0.00, 0.25]	[0.00, 0.29]	[0.71, 1.00]
Right anterior PFC academic	*r*	.19	.10	.60	*ACE*	0.18	0.02	0.80
	*p*	.16	.61		*95% CI*	[0.00, 0.42]	[0.00, 0.35]	[0.58, 1.00]
Right anterior PFC social	*r*	−.07	.26	−2.17^*^	*ACE*	0.00	0.08	0.92
	*p*	.59	.12		*95% CI*	[0.00, 0.26]	[0.00, 00.28]	[0.72, 1.00]
Dorsal mPFC academic	*r*	.10	−.13	1.49	*ACE*	0.05	0.00	0.95
	*p*	.46	.46		*95% CI*	[0.00, 0.30]	[0.00, 0.21]	[0.70, 1.00]
Dorsal mPFC social	*r*	.10	−.04	.91	*ACE*	0.07	0.00	0.93
	*p*	.53	.82		*95% CI*	[0.00, 0.21]	[0.00, 0.23]	[0.69, 1.00]
**Positive self > Negative self**								
mPFC (Denny et al., 2012) academic	*r*	.14	−.38	3.50^*^	*ACE*	0.00	0.00	1.00
	*p*	.32	<.05		*95% CI*	[0.00, 0.16]	[0.00, 0.10]	[0.84, 1.00]
mPFC (Denny et al., 2012) social	*r*	−.01	.13	−.91	*ACE*	0.00	0.00	1.00
	*p*	.96	.47		*95% CI*	[0.00, 0.17]	[0.00, 0.13]	[0.83, 1.00]
PCC academic	*r*	.02	.03	−.06	*ACE*	0.00	0.08	0.92
	*p*	.87	.88		*95% CI*	[0.00, 0.24]	[0.00, 0.22]	[0.76, 1.00]
PCC social	*r*	−.10	−.02	−0.52	*ACE*	0.00	0.00	1.00
	*p*	.58	.89		*95% CI*	[0.00, 0.18]	[0.00, 0.14]	[0.82, 1.00]
Ventral subg mPFC academic	*r*	−.10	.28	−2.21^*^	*ACE*	0.00	0.06	0.94
	*p*	.45	.09		*95% CI*	[0.00, 0.24]	[0.00, 0.26]	[0.76, 1.00]
Ventral subg mPFC social	*r*	.07	−.02	1.76^*^	*ACE*	0.05	0.00	0.95
	*p*	.59	.91		*95% CI*	[0.00, 0.32]	[*NA*, 0.24]	[0.66, 1.00]
**Academic > Social**								
Left DLPFC	*r*	−.10	.04	−.91	*ACE*	0.00	0.00	1.00
	*p*	.52	0.82		*95% CI*	[*NA*, 0.18]	[0.00, 0.16]	[0.81, 1.00]
Right DLPFC	*r*	.03	−.05	.52	*ACE*	0.00	0.00	1.00
	*p*	.81	.76		*95% CI*	[0.00, 0.24]	[*NA*, 0.20]	[0.76, 1.00]
**Social > Academic**								
mPFC (Denny et al., 2012)	*r*	.02	.12	−.65	*ACE*	0.00	0.00	1.00
	*p*	.89	.49		*95% CI*	[0.00, 0.18]	[0.00, 0.15]	[0.82, 1.00]
mPFC	*r*	.21	−.14	2.29^*^	*ACE*	0.13	0.00	0.87
	*p*	.13	.44		*95% CI*	[0.00, 0.36]	[0.00, 0.25]	[0.64, 1.00]

Abbreviations: A, additive genetic; C, shared environment; CI, confidence interval; DZ, dizygotic; E, unique environment/measurement error; MZ, monozygotic; *NA*, not available: the model was not able to calculate the CI; *p*, *p*‐value of significance; *r*, Pearson correlation; Z, test statistic z, significant Z‐scores indicate significant difference between MZ and DZ correlations.

^*^ *p* < .05.

Next, we used structural equation ACE modeling to reveal genetic, shared environment and unique environment driven effects (see Figure 5 for selected ROIs). Analyses are organized by contrast and separated by the independent and data‐driven ROI approach. Given the differential genetic and environmental contributions of academic and social self‐evaluations, all estimations are separated for academic and social traits.

#### 3.4.1 Independent mPFC ROI

ACE modeling was performed to explain the variation of mPFC activity, using the independent mPFC ROI (Denny et al., 2012), by heritability estimates in the *Self* > *Control*, *Positive Self* > *Negative Self*, and *Social* > *Academic* contrasts.

##### Self > Control

Variation in medial PFC activity for academic traits was accounted for by a combination of genetic factors and unique environment/measurement error (A = 14% and E = 86%). For social traits, variation in medial PFC activity was accounted for by shared environment and unique environment/measurement error (C = 17% and E = 83%).

##### Positive > Negative

All variation of medial PFC activity in both academic and social trials were accounted for by unique environment/measurement error.

##### Social > Academic

ACE modeling indicated that differences in neural activity in medial PFC for academic and social trials was all accounted for by unique environment/measurement error.

#### 3.4.2 Data‐driven ROIs

##### Self > Control

mPFC activity for academic traits was accounted for by a combination of shared environment (C = 7%) and unique environment/measurement error (E = 93%). For social trials, all variation in the mPFC was accounted for by unique environment/measurement error.

##### Negative > Positive

ACE modeling was performed for three ROIs. Variation in left lateral PFC activity was partly explained by shared environmental factors (academic: C = 8%, social: C = 9%). The remaining variation was accounted for by unique environment/measurement error, with no influence of genetics. For the anterior PFC variation in activity related to academic traits was 18% accounted for by genetic influence and 2% by shared environment influences (A = 18% and C = 2%). The remaining variation was explained for by unique environment/measurement error (E = 80%). For social trials, variation in anterior PFC was accounted for 8% by shared environment and 92% unique environment/measurement error (C = 8% and E = 92%). For dorsal medial PFC, variation in both academic and social trials were partly explained by genetic factors (academic: A = 5% and E = 95%, social: A = 7% and E = 93%).

##### Positive > Negative

ACE modeling indicated that variation in neural activity for PCC was mainly accounted for by unique environment/measurement error and shared environment (academic: C = 8% and E = 92%, social: E = 100%). For ventral subgenual mPFC, the activation during academic trials was accounted for 6% by shared environment influences (C = 6% and E = 92%). For social trials, activation in ventral subgenual mPFC was mainly accounted for by unique environment/measurement error (A = 5% and E = 95%).

##### Academic > Social

ACE modeling indicated that variation in neural activity in left and right DLPFC for academic trials were only accounted for by unique environment/measurement error (E = 100%).

##### Social > Academic

ACE modeling indicated that differences in neural activity in medial PFC for social trials was accounted for by a small percentage of genetic influence and mainly unique environment/measurement error (A = 13% and E = 87%).

### 3.5 Exploratory brain‐behavior associations

**FIGURE 6:**
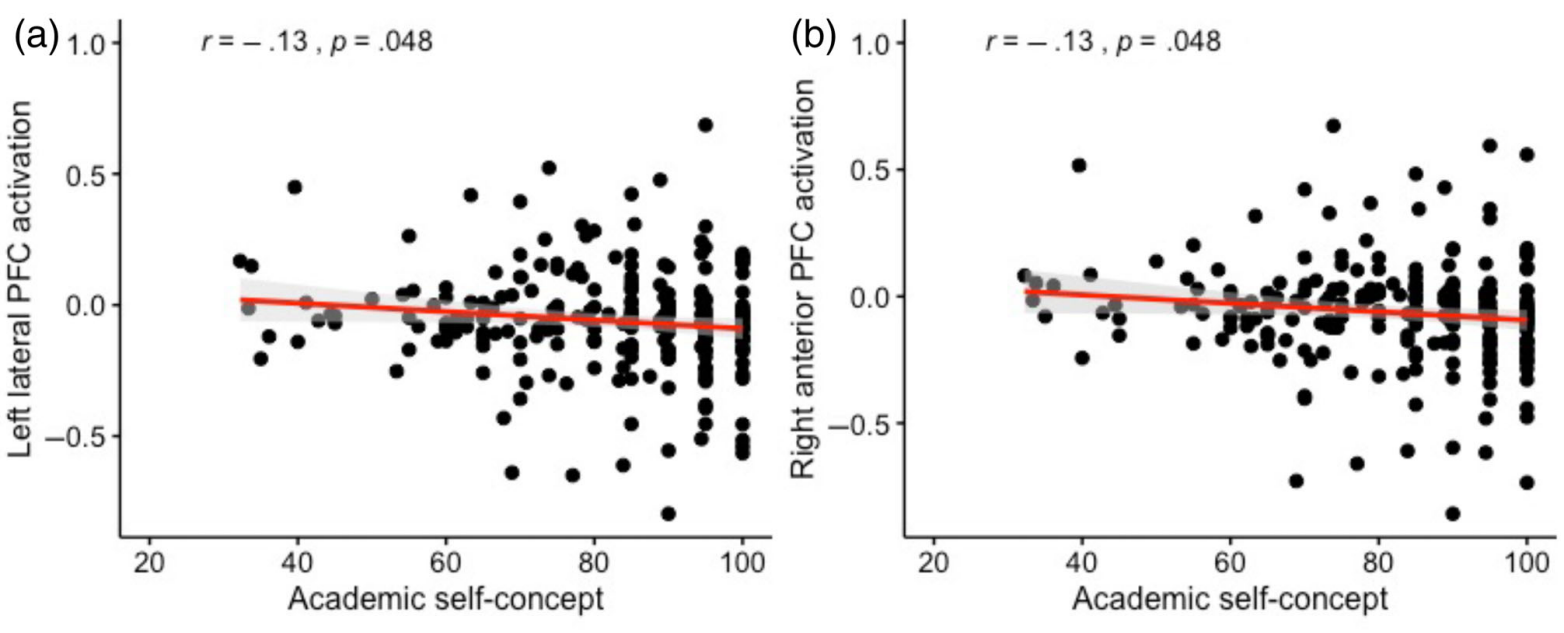
Brain‐behavior associations between academic self‐concept and two ROI's in the *Negative Self > Positive Self* contrast. (a) Lower academic self‐concept scores were correlated with increased activation in left lateral PFC. (b) Lower academic self‐concept scores were correlated with increased activation in right anterior PFC

To test for possible brain‐behavior associations, the behavioral positivity scores in the social and academic domain were correlated to the activity of three related ROIs using six Pearson correlations. The ROIs were extracted from the *Negative Self > Positive Self* contrast (left lateral PFC, right anterior PFC, and dorsal mPFC), see Figure 5. We observed two significant associations: (a) between academic positivity and left lateral PFC (*r*(230) = −.13, *p =* .048) and (b) between academic positivity and right anterior PFC (*r*(229) = −.13, *p* = .048), see Figure 6. These associations showed that higher positivity scores in the academic domain were related to less activation in both left lateral PFC and right anterior PFC. However, these associations did not survive Bonferroni correction (α *<* .01) for multiple testing and should be replicated in future research. Given that the two significant brain‐behavior correlations were identical, we performed another Pearson correlation between the activity in left lateral PFC and right anterior PFC. Indeed, activity in these brain regions were significantly correlated *r*(230) = .85, *p* = <.001. We did not observe significant brain‐behavior associations between dorsal mPFC and academic positivity nor between the three ROIs and social positivity. Furthermore, no significant brain‐behavior associations were observed in our whole‐brain regression analyses.

## 4 DISCUSSION

The aim of this study was to explore how neural correlates and behavioral aspects of self‐evaluations were influenced by genetic and environmental factors in middle childhood. This is the first study demonstrating in a young twin sample that behavioral and neural self‐concept depends on both genetic and shared environmental factors. Behaviorally, genetic modeling on 166 complete twin pairs revealed that social self‐evaluations were mainly explained by shared environment, whereas academic self‐evaluations were mainly explained by genetic factors. Our neural results are consistent with prior self‐processing activation results in adolescents and adults showing a strong contribution in mPFC for self‐concept generally (Moran et al., 2006; van der Cruijsen et al., 2018), more so for social self‐concept (van der Cruijsen et al., 2018) and for positive self‐concept (Denny et al., 2012; Northoff et al., 2006; van der Meer et al., 2010). In contrast, lateral PFC was most strongly related to academic (van der Cruijsen et al., 2018) and negative self‐concept.

In line with prior work and our expectations we observed increased activation in the cortical midline structures during self‐evaluations of 7–9‐year‐old children (*n* = 234; Denny et al., 2012; Northoff et al., 2006). Specifically, for self‐evaluations versus control trials, we confirmed increased mPFC activation in adolescents (van der Cruijsen et al., 2018) and adults (Denny et al., 2012; Northoff et al., 2006; van der Meer et al., 2010), indicating early development of brain regions underlying self‐evaluations that are already active in middle childhood. This neural evidence adds to the behavioral literature, reporting that middle childhood is an important period phase for children to start engaging in social behavior and social integration (DelGiudice, 2018) in which perspectives of others become more important in the forming of one's self‐concept (Harter, 2012). Moreover, a well‐balanced self‐concept has a positive effect on social functioning since a positive self‐concept was found to be an important factor for adjustment and for protection against problem behavior (Ybrandt, 2008).

Valence‐specific self‐evaluations elicited profound activation in the ventral subgenual mPFC for positive versus negative self traits, confirming reported findings in adolescents (van der Cruijsen et al., 2018) and adults (Moran et al., 2006). The ventral mPFC is anatomically situated in the medial orbital gyrus which is a region associated to positive valuation processes in adults (Kringelbach & Rolls, 2004; Peters & Büchel, 2010). Furthermore, we found increased activation in the PCC for positive evaluations which is suggested to be involved in autobiographical memory retrieval (Fink et al., 1996; Northoff & Bermpohl, 2004; Pfeifer & Peake, 2012; van der Cruijsen et al., 2018). In prior studies, this region is mainly observed during general and academic self‐evaluations in adolescents and adults (Moran et al., 2006; van der Cruijsen et al., 2018). Furthermore, given that the mPFC and PCC are also part of the DMN that is involved in self‐reference (Gusnard et al., 2001), adolescent and adult studies have associated increased activation in these DMN regions with negative emotions, such as depressive rumination (Zhou et al., 2020). This is not in line with our results. Possibly, these regions are more strongly engaged in positive self‐evaluations specifically in early and middle childhood, a period that is also characterized as a time of positivity bias (Harter, 2012; Trzesniewski, Donnellan, & Robins, 2003). Future studies including multiple age groups in a single design should test this potential specificity of mPFC and PCC for positive versus negative emotions.

Interestingly, for negative relative to positive self‐evaluations, we observed heightened activations in regions that are more commonly associated with cognitive control, including the right anterior PFC, left lateral PFC, and dorsal mPFC (Crone & Steinbeis, 2017). Others suggested that these regions are also important for memory retrieval and problem solving (Ramnani & Owen, 2004), self‐monitoring and self‐ versus other‐referential evaluations (Denny et al., 2012; Mitchell, Macrae, & Banaji, 2006; Murray, Schaer, & Debbané, 2012), and specifically dorsal mPFC to social rejection (Achterberg, van Duijvenvoorde, Bakermans‐Kranenburg, & Crone, 2016). Possibly, children might actively regulate emotional reactions in the PFC regions when they think about themselves in terms of negative traits (Silvers et al., 2016; Silvers & Moreira, 2019). Follow up brain‐behavior associations were analyzed to investigate this exploratory hypothesis. Left lateral PFC and right anterior PFC activity were predicted by academic self‐concept, showing that lower academic self‐concept was associated with increased activation in the PFC regions when evaluating negative traits, although these associations did not survive Bonferroni correction.

Furthermore, the present study showed domain‐specific neural activation for academic and social traits. We followed upon the results of adolescent (van der Cruijsen et al., 2018) and adult (Moran et al., 2006) studies, in which academic self‐evaluations elicited more bilateral DLPFC activation compared to social self‐evaluations. The DLPFC regions are often related to processes of semantic memory retrieval (Badre & Wagner, 2007; Martinelli, Sperduti, & Piolino, 2013; Thompson‐Schill, Bedny, & Goldberg, 2005). When evaluating social versus academic traits, children engaged the mPFC suggesting that this region is mainly important in forming social self‐concept. We showed that children reacted slower to academic versus social self‐evaluations. This can indicate that academic self‐evaluations take longer to process suggesting a more difficult judgment. Apparently, academic self‐concept is more difficult to judge in middle childhood whereas social self‐concept is suggestively more difficult to judge in puberty and adolescence. Possible explanations are the period of positivity bias (Trzesniewski et al., 2003) and the increased ratings children receive on an academic level (e.g., grades in report) compared to the ratings children receive on a social level where they still compare themselves with themselves in the past rather than with their social environment (Harter, 2012). Additionally, task difficulty could be a potential contributor to differences in brain activation between the academic and social domain. As such, bilateral DLPFC activation may be partly explained by more difficult in judging academic self‐concept in 7–9‐year‐olds (Tregellas, Davalos, & Rojas, 2006).

Given the differential genetic and environmental contributions of academic and social self‐evaluations on a behavioral level, we investigated whether we could unravel similar domain‐specific effects in neural activity. Therefore, heritability estimates were separated for academic and social traits on 91 complete twin pairs. Two specific neural findings confirmed the domain‐specific effect in heritability estimates for self‐evaluations relative to control trials and negative relative to positive self‐evaluations; mPFC (based on the independent ROI of Denny et al., 2012) and right anterior PFC activity variation related to *academic* traits was partly explained by genetic factors, whereas mPFC and right anterior PFC activity variation related to *social* traits was partly explained by shared environmental factors. Intriguingly, mPFC which is one of the key regions for processing oneself shows either genetic or social environment influences depending on the domain. Prior studies have interpreted mPFC as a hub region when processing information related to self and others (Crone & Fuligni, 2020). Possibly, mPFC is functionally connected to separable networks, such as the genetically influenced DMN (Glahn et al., 2010) and social salience network (Achterberg, Van Duijvenvoorde, van IJzendoorn, Bakermans‐Kranenburg, & Crone, 2020), depending on the domain that is targeted. Prior studies that examined structural development of the mPFC showed strong influences of genetic estimates on mPFC cortical thickness (van der Meulen et al., 2020), whereas others showed that cortical thickness development is influenced by friendship experiences (Becht et al., 2021).

Since behavioral and neural genetic modeling revealed domain‐specific heritability estimates in the academic domain, this suggests that intelligence might have driven the genetic component in academic self‐concept. Prior work showed that intelligence is a strong predictor of educational achievement and highly heritable (30%; Deary, Johnson, & Houlihan, 2009). Moreover, prior studies have demonstrated the mutual reinforcement between academic self‐concept and academic achievement, each leading to gains in the other (Marsh & Martin, 2011). However, performance IQ measured with subset “Picture Completion” (Preschool, 2002), did not predict academic self‐concept in our study. For future research, the Verbal subset including subsets as “Vocabulary,” “Information,” and “Word Reasoning” (Preschool, 2002) should also be used to measure intelligence in children since language is an essential component of educational achievement (Spinath, Freudenthaler, & Neubauer, 2010). Furthermore, academic self‐concept and intelligence may be separable constructs that influence each other but are dissociable (van der Aar, Peters, van der Cruijsen, & Crone, 2019). Another interpretation of the domain‐specific heritability estimates could be that cognitive ability is more involved in evaluating one's academic self (Bong & Skaalvik, 2003), whereas social environment is more involved in evaluating one's social self. In line with this possibility, prior studies reported that cognitive abilities are substantially influenced by genes (Haworth et al., 2010), whereas social behavior is mostly influenced by environmental factors (van der Meulen et al., 2018).

Regardless of domain‐specificity, we found that for negative self‐evaluations variation in both lateral PFC and anterior PFC activation was partly explained by shared environment and with a small percentage by genetic influences. Of note, the confidence intervals include zero and therefore the results need to be interpreted with caution. Suggestively, the more standing out influence of shared environment, such as parents, might be important for neural responses to negative compared to positive self‐evaluations. Children with parents who are neglectful or use excessively harsh punishment, are less able to develop a sense of themselves as loveable and competent (Bowlby, 1982) and are more likely to perceive themselves as unworthy (Kim & Cicchetti, 2010; Turner, Finkelhor, & Ormrod, 2006). In line with these findings, additional behavioral studies have reported more negative self‐concepts in maltreated than nonmaltreated children indicating the large impact of negative environment on self‐concept (Bolger, Patterson, & Kupersmidt, 1998; Kim & Cicchetti, 2010; Toth, Cicchetti, MacFie, Maughan, & Vanmeenen, 2000). Our work presents the first indication of the mixed nature of genetic and environmental effects on neural responses to self‐evaluations. Future research is needed to further explore heritability analyses on fMRI task‐based self‐concept for comparisons of these results and to investigate *which* specific genetic and environmental factors are key in explaining the variance of self‐concept in middle childhood.

Several strengths of this study should be addressed including the robustness of our large twin study (*n* = 345) and the confirmed neural correlates of meta‐analytic studies regarding self‐concept in adolescents and adults. Furthermore, this is the first study examining heritability estimates of task‐based fMRI self‐concept in middle childhood. However, several limitations of this study should also be brought to light for future directions. It remains unclear whether our self‐concept fMRI paradigm is test–retest reliable since not enough trials were included to examine the intraclass correlations (Elliott et al., 2020). Although our sample size is considered large with respect to fMRI, it is relatively small with regards to statistical power of genetic modeling (Verhulst, 2017). As such, the heritability estimates of the neural ROI results should be interpreted with caution. The confidence intervals of the genetic and environmental contributions must be taken into account since all contain zero. Last, in the current study we observed children in a small age range. Longitudinal designs are needed to visualize changes in heritability estimates with a focus on the transition between childhood and adolescence as in this period peers, opinions of others and higher expectations of academic achievement become increasingly important (Chung et al., 2014). Nevertheless, this study provides innovative heritability findings which can be further explored in future studies.

Taken together, our results highlight the significant domain‐specific effects of genetic and environmental inputs on the observed behavior and neural correlates of self‐concept in middle childhood, with stronger environmental influences in the social versus academic domain. Therefore, this study implies possibilities for behavioral interventions situated in the (social) environment, such as parenting programs, specifically aimed at improving social self‐concept of the child. Parents who are suggested to make the child feel competent, can contribute to a more positive self‐concept of the child and furthermore a belonging in the social world (Bracken, 2009). Additionally, neural evidence of activation patterns in the cortical midline structures and PFC regions fit with prior research in adolescents (van der Cruijsen et al., 2018) and adults (Denny et al., 2012; Moran et al., 2006; Murray et al., 2012). Our neural results are the first building blocks for understanding the early trajectory of self‐concept, which may lead the way to more extensive studies on genetic and environmental effects on fMRI task‐based self‐concept development.

## CONFLICT OF INTERESTS

The authors declare no competing financial interests.

## AUTHOR CONTRIBUTIONS

Lina van Drunen and Simone Dobbelaar collected behavioral and fMRI data. Lina van Drunen with assistance of Renske van der Cruijsen designed the self‐concept fMRI paradigm. Lina van Drunen with assistance of Michelle Achterberg and Mara van der Meulen performed and analyzed genetic modeling. Lina van Drunen with assistance of Lara M. Wierenga designed the analysis script in the software R. Lina van Drunen and Eveline A. Crone designed research, analyzed data, and wrote article with edit contributions of all co‐authors.

## ETHICS STATEMENT

The study and procedures were approved by the Dutch Central Committee on Research Involving Human Subjects (CCMO).

## Data Availability

Data that support the findings of this study will become available after publication in DataverseNL at https://doi.org/10.34894/IZ35Z1. Group‐level MRI data will be available in NeuroVault after publication. During the review process, the data is available upon request with the corresponding author.

## References

[hbm25641-bib-0001] Achterberg, M. , & van der Meulen, M. (2019). Genetic and environmental influences on MRI scan quantity and quality. Developmental Cognitive Neuroscience, 38, 100667. 31170550 10.1016/j.dcn.2019.100667 PMC6969338

[hbm25641-bib-0002] Achterberg, M. , van Duijvenvoorde, A. C. K. , Bakermans‐Kranenburg, M. J. , & Crone, E. A. (2016). Control your anger! The neural basis of aggression regulation in response to negative social feedback. Social Cognitive and Affective Neuroscience, 11(5), 712–720. 26755768 10.1093/scan/nsv154 PMC4847693

[hbm25641-bib-0003] Achterberg, M. , Van Duijvenvoorde, A. C. K. , van der Meulen, M. , Bakermans‐Kranenburg, M. J. , & Crone, E. A. (2018). Heritability of aggression following social evaluation in middle childhood: An fMRI study. Human Brain Mapping, 39(7), 2828–2841. 29528161 10.1002/hbm.24043 PMC6055731

[hbm25641-bib-0004] Achterberg, M. , Van Duijvenvoorde, A. C. K. , van IJzendoorn, M. H. , Bakermans‐Kranenburg, M. J. , & Crone, E. A. (2020). Longitudinal changes in DLPFC activation during childhood are related to decreased aggression following social rejection. Proceedings of the National Academy of Sciences, 117(15), 8602–8610. 10.1073/pnas.1915124117 PMC7165424 32234781

[hbm25641-bib-0005] Amodio, D. M. , & Frith, C. D. (2006). Meeting of minds: The medial frontal cortex and social cognition. Nature Reviews Neuroscience, 7(4), 268–277. 16552413 10.1038/nrn1884

[hbm25641-bib-0006] Badre, D. , & Wagner, A. D. (2007). Left ventrolateral prefrontal cortex and the cognitive control of memory. Neuropsychologia, 45(13), 2883–2901. 17675110 10.1016/j.neuropsychologia.2007.06.015

[hbm25641-bib-0007] Barendse, M. E. A. , Cosme, D. , Flournoy, J. C. , Vijayakumar, N. , Cheng, T. W. , Allen, N. B. , & Pfeifer, J. H. (2020). Neural correlates of self‐evaluation in relation to age and pubertal development in early adolescent girls. Developmental Cognitive Neuroscience, 44, 100799. 32479376 10.1016/j.dcn.2020.100799 PMC7260676

[hbm25641-bib-0008] Becht, A. I. , Wierenga, L. M. , Mills, K. L. , Meuwese, R. , van Duijvenvoorde, A. , Blakemore, S.‐J. , … Crone, E. A. (2021). Beyond the average brain: Individual differences in social brain development are associated with friendship quality. Social Cognitive and Affective Neuroscience, 16(3), 292–301. 33277895 10.1093/scan/nsaa166 PMC7943358

[hbm25641-bib-0009] Bolger, K. E. , Patterson, C. J. , & Kupersmidt, J. B. (1998). Peer relationships and self‐esteem among children who have been maltreated. Child Development, 69(4), 1171–1197. 9768492

[hbm25641-bib-0010] Bong, M. , & Skaalvik, E. M. (2003). Academic self‐concept and self‐efficacy: How different are they really? Educational Psychology Review, 15(1), 1–40.

[hbm25641-bib-0011] Bowlby, J. (1982). Attachment and loss: Retrospect and prospect. American Journal of Orthopsychiatry, 52(4), 664. 10.1111/j.1939-0025.1982.tb01456.x 7148988

[hbm25641-bib-0012] Bracken, B. A. (2009). Positive self‐concepts. In Handbook of Positive Psychology in Schools (pp. 89–106).Londen, United Kingdom: Routledge.

[hbm25641-bib-0013] Brett, M. , Anton, J.‐L. , Valabregue, R. , & Poline, J.‐B. (2002). Region of interest analysis using the MarsBar toolbox for SPM 99. NeuroImage, 16(2), S497.

[hbm25641-bib-0014] Chung, J. M. , Robins, R. W. , Trzesniewski, K. H. , Noftle, E. E. , Roberts, B. W. , & Widaman, K. F. (2014). Continuity and change in self‐esteem during emerging adulthood. Journal of Personality and Social Psychology, 106(3), 469. 24377355 10.1037/a0035135 PMC4049296

[hbm25641-bib-0015] Cocosco, C. A. , Kollokian, V. , Kwan, R. K.‐S. , Pike, G. B. & Evans, A. C. (1997). Brainweb: Online interface to a 3D MRI simulated brain database. NeuroImage. Citeseer.

[hbm25641-bib-0016] Crone E. A. Achterberg M. Dobbelaar S. Euser S. van den Bulk B. , , & (2020). Neural and behavioral signatures of social evaluation and adaptation in childhood and adolescence: The Leiden consortium on individual development (L‐CID). Developmental Cognitive Neuroscience, 100805. 10.1016/j.dcn.2020.100805 PMC7390777 33040969

[hbm25641-bib-0017] Crone, E. A. , & Fuligni, A. J. (2020). Self and others in adolescence. Annual Review of Psychology, 71, 447–469. 10.1146/annurev-psych-010419-050937 31337274

[hbm25641-bib-0018] Crone, E. A. , & Steinbeis, N. (2017). Neural perspectives on cognitive control development during childhood and adolescence. Trends in Cognitive Sciences, 21(3), 205–215. 28159355 10.1016/j.tics.2017.01.003

[hbm25641-bib-0019] Dale, A. M. (1999). Optimal experimental design for event‐related fMRI. Human Brain Mapping, 8(2–3), 109–114. 10524601 10.1002/(SICI)1097-0193(1999)8:2/3<109::AID-HBM7>3.0.CO;2-W PMC6873302

[hbm25641-bib-0020] Davey, C. G. , Pujol, J. , & Harrison, B. J. (2016). Mapping the self in the brain's default mode network. NeuroImage, 132, 390–397. 26892855 10.1016/j.neuroimage.2016.02.022

[hbm25641-bib-0021] Deary, I. J. , Johnson, W. , & Houlihan, L. M. (2009). Genetic foundations of human intelligence. Human Genetics, 126(1), 215–232. 19294424 10.1007/s00439-009-0655-4

[hbm25641-bib-0022] DelGiudice, M. (2018). Middle childhood: An evolutionary‐developmental synthesis. In Handbook of life course health development (pp. 95–107). Cham: Springer. 31314298

[hbm25641-bib-0023] Denny, B. T. , Kober, H. , Wager, T. D. , & Ochsner, K. N. (2012). A meta‐analysis of functional neuroimaging studies of self‐ and other judgments reveals a spatial gradient for mentalizing in medial prefrontal cortex. Journal of Cognitive Neuroscience, 24(8), 1742–1752. 10.1162/jocn_a_00233 22452556 PMC3806720

[hbm25641-bib-0024] Elliott, M. L. , Knodt, A. R. , Ireland, D. , Morris, M. L. , Poulton, R. , Ramrakha, S. , … Hariri, A. R. (2020). What is the test‐retest reliability of common task‐functional MRI measures? New empirical evidence and a meta‐analysis. Psychological Science, 31(7), 792–806. 32489141 10.1177/0956797620916786 PMC7370246

[hbm25641-bib-0025] Euser, S. , Bakermans‐Kranenburg, M. J. , van den Bulk, B. G. , Linting, M. , Damsteegt, R. C. , Vrijhof, C. I. , … van IJzendoorn, M.H. (2016). Efficacy of the video‐feedback intervention to promote positive parenting and sensitive discipline in twin families (VIPP‐twins): Study protocol for a randomized controlled trial. BMC Psychology, 4(1), 1–11. 27268415 10.1186/s40359-016-0139-y PMC4895801

[hbm25641-bib-0026] Fink, G. R. , Markowitsch, H. J. , Reinkemeier, M. , Bruckbauer, T. , Kessler, J. , & Heiss, W.‐D. (1996). Cerebral representation of one's own past: Neural networks involved in autobiographical memory. Journal of Neuroscience, 16(13), 4275–4282. 8753888 10.1523/JNEUROSCI.16-13-04275.1996 PMC6579004

[hbm25641-bib-0027] Glahn, D. C. , Winkler, A. M. , Kochunov, P. , Almasy, L. , Duggirala, R. , Carless, M. A. , … Smith, S. M. (2010). Genetic control over the resting brain. Proceedings of the National Academy of Sciences, 107(3), 1223–1228. 10.1073/pnas.0909969107 PMC2824276 20133824

[hbm25641-bib-0028] Gusnard, D. A. , Akbudak, E. , Shulman, G. L. , & Raichle, M. E. (2001). Medial prefrontal cortex and self‐referential mental activity: Relation to a default mode of brain function. Proceedings of the National Academy of Sciences, 98(7), 4259–4264. 10.1073/pnas.071043098 PMC31213 11259662

[hbm25641-bib-0029] Gusnard, D. A. , & Raichle, M. E. (2001). Searching for a baseline: Functional imaging and the resting human brain. Nature Reviews Neuroscience, 2(10), 685–694. 11584306 10.1038/35094500

[hbm25641-bib-0030] Harter, S. (1988). Self‐perception profile for adolescents. Denver, Colorado: University of Denver.

[hbm25641-bib-0031] Harter, S. (2012). The construction of the self: Developmental and sociocultural foundations. In The construction of the self: Developmental and sociocultural foundations (2nd ed.). New York, NY: The Guilford Press.

[hbm25641-bib-0032] Haworth, C. M. A. , Wright, M. J. , Luciano, M. , Martin, N. G. , de Geus, E. J. C. , van Beijsterveldt, C. E. M. , … Davis, O. S. P. (2010). The heritability of general cognitive ability increases linearly from childhood to young adulthood. Molecular Psychiatry, 15(11), 1112–1120. 19488046 10.1038/mp.2009.55 PMC2889158

[hbm25641-bib-0033] Hur, Y.‐M. , McGue, M. , & Iacono, W. G. (1998). The structure of self‐concept in female preadolescent twins: A behavioral genetic approach. Journal of Personality and Social Psychology, 74(4), 1069. 9569660 10.1037//0022-3514.74.4.1069

[hbm25641-bib-0034] Jankowski, K. F. , Moore, W. E. , Merchant, J. S. , Kahn, L. E. , & Pfeifer, J. H. (2014). But do you think I'm cool?: Developmental differences in striatal recruitment during direct and reflected social self‐evaluations. Developmental Cognitive Neuroscience, 8, 40–54. 24582805 10.1016/j.dcn.2014.01.003 PMC4422645

[hbm25641-bib-0035] Kim, J. , & Cicchetti, D. (2010). Longitudinal pathways linking child maltreatment, emotion regulation, peer relations, and psychopathology. Journal of Child Psychology and Psychiatry, 51(6), 706–716. 20050965 10.1111/j.1469-7610.2009.02202.x PMC3397665

[hbm25641-bib-0036] Kringelbach, M. L. , & Rolls, E. T. (2004). The functional neuroanatomy of the human orbitofrontal cortex: Evidence from neuroimaging and neuropsychology. Progress in Neurobiology, 72(5), 341–372. 15157726 10.1016/j.pneurobio.2004.03.006

[hbm25641-bib-0037] Legrand, D. , & Ruby, P. (2009). What is self‐specific? Theoretical investigation and critical review of neuroimaging results. Psychological Review, 116(1), 252. 19159156 10.1037/a0014172

[hbm25641-bib-0038] Lieberman, M. D. , Straccia, M. A. , Meyer, M. L. , Du, M. , & Tan, K. M. (2019). Social, self (situational), and affective processes in medial prefrontal cortex (MPFC): Causal, multivariate, and reverse inference evidence. Neuroscience & Biobehavioral Reviews, 99, 311–328. 30610911 10.1016/j.neubiorev.2018.12.021

[hbm25641-bib-0039] Marsh, H. W. , & Ayotte, V. (2003). Do multiple dimensions of self‐concept become more differentiated with age? The differential distinctiveness hypothesis. Journal of Educational Psychology, 95(4), 687.

[hbm25641-bib-0040] Marsh, H. W. , & Martin, A. J. (2011). Academic self‐concept and academic achievement: Relations and causal ordering. British Journal of Educational Psychology, 81(1), 59–77. 10.1348/000709910X503501 21391964

[hbm25641-bib-0041] Martinelli, P. , Sperduti, M. , & Piolino, P. (2013). Neural substrates of the self‐memory system: New insights from a meta‐analysis. Human Brain Mapping, 34(7), 1515–1529. 22359397 10.1002/hbm.22008 PMC6870171

[hbm25641-bib-0042] Mason, M. F. , Norton, M. I. , Van Horn, J. D. , Wegner, D. M. , Grafton, S. T. , & Macrae, C. N. (2007). Wandering minds: The default network and stimulus‐independent thought. Science, 315(5810), 393–395. 17234951 10.1126/science.1131295 PMC1821121

[hbm25641-bib-0043] Mitchell, J. P. , Macrae, C. N. , & Banaji, M. R. (2006). Dissociable medial prefrontal contributions to judgments of similar and dissimilar others. Neuron, 50(4), 655–663. 16701214 10.1016/j.neuron.2006.03.040

[hbm25641-bib-0044] Moran, J. M. , Macrae, C. N. , Heatherton, T. F. , Wyland, C. L. , & Kelley, W. M. (2006). Neuroanatomical evidence for distinct cognitive and affective components of self. Journal of Cognitive Neuroscience, 18(9), 1586–1594. 16989558 10.1162/jocn.2006.18.9.1586

[hbm25641-bib-0045] Muris, P. , Meesters, C. , & van den Berg, S. (2003). Internalizing and externalizing problems as correlates of self‐reported attachment style and perceived parental rearing in normal adolescents. Journal of Child and Family Studies, 12(2), 171–183.

[hbm25641-bib-0046] Murray, R. J. , Schaer, M. , & Debbané, M. (2012). Degrees of separation: A quantitative neuroimaging meta‐analysis investigating self‐specificity and shared neural activation between self‐and other‐reflection. Neuroscience & Biobehavioral Reviews, 36(3), 1043–1059. 22230705 10.1016/j.neubiorev.2011.12.013

[hbm25641-bib-0047] Neale, M. C. , Hunter, M. D. , Pritikin, J. N. , Zahery, M. , Brick, T. R. , Kirkpatrick, R. M. , … Boker, S. M. (2016). OpenMx 2.0: Extended structural equation and statistical modeling. Psychometrika, 81(2), 535–549. 25622929 10.1007/s11336-014-9435-8 PMC4516707

[hbm25641-bib-0048] Neiss, M. B. , Sedikides, C. , & Stevenson, J. (2002). Self‐esteem: A behavioural genetic perspective. European Journal of Personality, 16(5), 351–367.

[hbm25641-bib-0049] Northoff, G. , & Bermpohl, F. (2004). Cortical midline structures and the self. Trends in Cognitive Sciences, 8(3), 102–107. 15301749 10.1016/j.tics.2004.01.004

[hbm25641-bib-0050] Northoff, G. , Heinzel, A. , De Greck, M. , Bermpohl, F. , Dobrowolny, H. , & Panksepp, J. (2006). Self‐referential processing in our brain—A meta‐analysis of imaging studies on the self. NeuroImage, 31(1), 440–457. 16466680 10.1016/j.neuroimage.2005.12.002

[hbm25641-bib-0051] Peters, J. , & Büchel, C. (2010). Neural representations of subjective reward value. Behavioural Brain Research, 213(2), 135–141. 20420859 10.1016/j.bbr.2010.04.031

[hbm25641-bib-0052] Pfeifer, J. H. , Lieberman, M. D. , & Dapretto, M. (2007). “I know you are but what am I?!”: Neural bases of self‐and social knowledge retrieval in children and adults. Journal of Cognitive Neuroscience, 19(8), 1323–1337. 17651006 10.1162/jocn.2007.19.8.1323 PMC3407805

[hbm25641-bib-0053] Pfeifer, J. H. , & Peake, S. J. (2012). Self‐development: Integrating cognitive, socioemotional, and neuroimaging perspectives. Developmental Cognitive Neuroscience, 2(1), 55–69. 22682728 10.1016/j.dcn.2011.07.012 PMC6987679

[hbm25641-bib-0054] Preschool, W. D. W. (2002). Primary scale of intelligence. San Antonio, TX: Psychological Corporation.

[hbm25641-bib-0055] Qin, P. , & Northoff, G. (2011). How is our self related to midline regions and the default‐mode network? NeuroImage, 57(3), 1221–1233. 21609772 10.1016/j.neuroimage.2011.05.028

[hbm25641-bib-0080] R Core Team. (2015). R: A language and environment for statistical computing. Vienna, Austria: R Foundation for Statistical Computing. https://www.R-project.org/

[hbm25641-bib-0056] Ramnani, N. , & Owen, A. M. (2004). Anterior prefrontal cortex: Insights into function from anatomy and neuroimaging. Nature Reviews Neuroscience, 5(3), 184–194. 14976518 10.1038/nrn1343

[hbm25641-bib-0057] Ray, R. D. , Shelton, A. L. , Hollon, N. G. , Michel, B. D. , Frankel, C. B. , Gross, J. J. , & Gabrieli, J. D. E. (2009). Cognitive and neural development of individuated self‐representation in children. Child Development, 80(4), 1232–1242. 19630904 10.1111/j.1467-8624.2009.01327.x PMC4138977

[hbm25641-bib-0058] Rochat, P. , & Striano, T. (2002). Who's in the mirror? Self‐other discrimination in specular images by four‐ and nine‐month‐old infants. Child Development, 73(1), 35–46. 10.1111/1467-8624.00390 14717242

[hbm25641-bib-0059] Schneider, W. , Eschman, A. , & Zuccolotto, A. (2002). E‐prime: User's guide. Inc., Pittsburgh, PA.: Psychology Software Incorporated.

[hbm25641-bib-0060] Sedikides, C. , & Skowronski, J. J. (1997). The symbolic self in evolutionary context. Personality and Social Psychology Review, 1(1), 80–102. 15647130 10.1207/s15327957pspr0101_6

[hbm25641-bib-0061] Silvers, J. A. , Hubbard, A. D. , Biggs, E. , Shu, J. , Fertuck, E. , Chaudhury, S. , … Chesin, M. (2016). Affective lability and difficulties with regulation are differentially associated with amygdala and prefrontal response in women with borderline personality disorder. Psychiatry Research: Neuroimaging, 254, 74–82. 27379614 10.1016/j.pscychresns.2016.06.009 PMC4992645

[hbm25641-bib-0062] Silvers, J. A. , & Moreira, J. F. G. (2019). Capacity and tendency: A neuroscientific framework for the study of emotion regulation. Neuroscience Letters, 693, 35–39. 28899785 10.1016/j.neulet.2017.09.017

[hbm25641-bib-0063] Spinath, B. , Freudenthaler, H. H. , & Neubauer, A. C. (2010). Domain‐specific school achievement in boys and girls as predicted by intelligence, personality and motivation. Personality and Individual Differences, 48(4), 481–486.

[hbm25641-bib-0064] Thompson‐Schill, S. L. , Bedny, M. , & Goldberg, R. F. (2005). The frontal lobes and the regulation of mental activity. Current Opinion in Neurobiology, 15(2), 219–224. 15831406 10.1016/j.conb.2005.03.006

[hbm25641-bib-0065] Toth, S. L. , Cicchetti, D. , MacFie, J. , Maughan, A. , & Vanmeenen, K. (2000). Narrative representations of caregivers and self in maltreated pre‐schoolers. Attachment & Human Development, 2(3), 271–305. 11708220 10.1080/14616730010000849

[hbm25641-bib-0066] Tregellas, J. R. , Davalos, D. B. , & Rojas, D. C. (2006). Effect of task difficulty on the functional anatomy of temporal processing. NeuroImage, 32(1), 307–315. 16624580 10.1016/j.neuroimage.2006.02.036

[hbm25641-bib-0067] Trzesniewski, K. H. , Donnellan, M. B. , & Robins, R. W. (2003). Stability of self‐esteem across the life span. Journal of Personality and Social Psychology, 84(1), 205. 12518980

[hbm25641-bib-0068] Turner, H. A. , Finkelhor, D. , & Ormrod, R. (2006). The effect of lifetime victimization on the mental health of children and adolescents. Social Science & Medicine, 62(1), 13–27. 16002198 10.1016/j.socscimed.2005.05.030

[hbm25641-bib-0069] van der Aar, L. P. E. , Peters, S. , van der Cruijsen, R. , & Crone, E. A. (2019). The neural correlates of academic self‐concept in adolescence and the relation to making future‐oriented academic choices. Trends in Neuroscience and Education, 15, 10–17. 10.1016/j.tine.2019.02.003 31176467

[hbm25641-bib-0070] van der Cruijsen, R. , Peters, S. , & Crone, E. A. (2017). Neural correlates of evaluating self and close‐other in physical, academic and prosocial domains. Brain and Cognition, 118, 45–53. 10.1016/j.bandc.2017.07.008 28759780

[hbm25641-bib-0071] van der Cruijsen, R. , Peters, S. , van der Aar, L. P. E. , & Crone, E. A. (2018). The neural signature of self‐concept development in adolescence: The role of domain and valence distinctions. Developmental Cognitive Neuroscience, 30, 1–12. 10.1016/j.dcn.2017.11.005 29197726 PMC6969085

[hbm25641-bib-0072] van der Meer, L. , Costafreda, S. , Aleman, A. , & David, A. S. (2010). Self‐reflection and the brain: A theoretical review and meta‐analysis of neuroimaging studies with implications for schizophrenia. Neuroscience & Biobehavioral Reviews, 34(6), 935–946. 20015455 10.1016/j.neubiorev.2009.12.004

[hbm25641-bib-0073] van der Meulen, M. , Steinbeis, N. , Achterberg, M. , van IJzendoorn, M.H. , & Crone, E. A. (2018). Heritability of neural reactions to social exclusion and prosocial compensation in middle childhood. Developmental Cognitive Neuroscience, 34, 42–52. 29936358 10.1016/j.dcn.2018.05.010 PMC6969304

[hbm25641-bib-0074] van der Meulen, M. , Wierenga, L. M. , Achterberg, M. , Drenth, N. , van IJzendoorn, M.H. , & Crone, E.A. (2020). Genetic and environmental influences on structure of the social brain in childhood. Developmental Cognitive Neuroscience, 44, 100782. 32716847 10.1016/j.dcn.2020.100782 PMC7374548

[hbm25641-bib-0075] van Overwalle, F. (2011). A dissociation between social mentalizing and general reasoning. NeuroImage, 54(2), 1589–1599. 20869452 10.1016/j.neuroimage.2010.09.043

[hbm25641-bib-0076] Verhulst, B. (2017). A power calculator for the classical twin design. Behavior Genetics, 47(2), 255–261. 27866285 10.1007/s10519-016-9828-9 PMC5471839

[hbm25641-bib-0077] Ybrandt, H. (2008). The relation between self‐concept and social functioning in adolescence. Journal of Adolescence, 31(1), 1–16. 17467050 10.1016/j.adolescence.2007.03.004

[hbm25641-bib-0078] Yuan, M. , Li, Y. , Yang, Y. , Xu, J. , Tao, F. , Zhao, L. , … Xu, X. S. (2020). A novel quantification of information for longitudinal data analyzed by mixed‐effects modeling. Pharmaceutical Statistics, 19, 388–398. 31989784 10.1002/pst.1996

[hbm25641-bib-0079] Zhou, H.‐X. , Chen, X. , Shen, Y.‐Q. , Li, L. , Chen, N.‐X. , Zhu, Z.‐C. , … Yan, C.‐G. (2020). Rumination and the default mode network: Meta‐analysis of brain imaging studies and implications for depression. NeuroImage, 206, 116287. 31655111 10.1016/j.neuroimage.2019.116287

